# Structured classification for ED presenting complaints – from free text field-based approach to ICPC-2 ED application

**DOI:** 10.1186/1757-7241-20-76

**Published:** 2012-11-24

**Authors:** Tomi Malmström, Olli Huuskonen, Paulus Torkki, Raija Malmström

**Affiliations:** 1 Institute of Healthcare Engineering and Management, Department of Industrial Engineering and Management, Aalto University, Otaniementie 17, 00076, Aalto, Finland; 2 Jorvi Hospital, Division of Emergency Care, Meilahti Hospital, Helsinki University Hospital District, Helsinki, Finland; 3 Department of Medicine, Division of Emergency Care, Meilahti Hospital, Helsinki University Hospital District, Helsinki, Finland

## Abstract

**Background:**

Although there is a major need to record and analyse presenting complaints in emergency departments (EDs), no international standard exists. The aim of the present study was to produce structured complaint classification suitable for ED use and to implement it in practice. The structured classification evolved from a study of free text fields and ICPC-2 classification.

**Methods:**

Presenting complaints in a free text field of ED admissions during a one-year period (n=40610) were analyzed and summarized to 70 presenting complaint groups. The results were compared to ICPC-2 based complaints collected in another ED. An expert panel reviewed the results and produced an ED application of ICPC-2 classification. This study implemented the new classification into an ED.

**Results:**

The presenting complaints summarized from free text fields and those from ICPC-2 categories were remarkably similar. However, the ICPC-2 classification was too broad for ED; an adapted version was needed. The newly developed classification includes 89 presenting complaints and ED staff found it easy to use.

**Conclusions:**

ICPC-2 classification can be adapted for ED use. The authors suggest a list of 89 presenting complaints for use in EDs adult patients.

## Background

When a patient enters an emergency department (ED), important decisions are made at the very beginning of the visit concerning the necessity for, and the urgency of, medical examinations and care required by the patient. The presenting complaint, other anamnesis available, and a short status assessment determines the urgency of the treatment. Information from the referral notes or ambulance staff is also useful, if available. The presenting complaint is the patient’s reason for the encounter, interpreted and recorded by the triage nurse. Although the importance of triage on patients’ prognoses is recognized [1], there exists only sparse data on the presenting complaints of ED patients.

Over the last 20 years, hospitals have developed different formal triage systems and these systems are in wide use [1]. No golden standard for triage exists, and careful attention should be paid on the studies, and the follow-ups, of the competence and validity of the triage process in each ED. For example, studies on children [2], patients with unspecific complaints [3], and patients with sepsis [4] have shown the difficulties of triage.

To be able to study the impact of triage on the prognoses of different patient groups, structured information of the presenting complaints is needed. The process, from presenting complaint to diagnosis, is the core competence of emergency departments and failures at the beginning of the process - specifically, in triage - often lead to prolonged visits, endangered patient safety, and decreased patient satisfaction. Designing and controlling emergency departments with end diagnosis based information may result in inappropriate processes. A system that allows EDs to classify patients and define comparable case-mix groupings will help EDs describe their patient populations, workloads, staffing, and resource needs, and enable comparison across sites and regions [5]. In addition, it allows the development of automated decision support systems to specific patient groups, such as the automated evaluation of the Pneumonia Severity Index [6], or the reminder of stroke assessment form [7]. However, such a classification system should be relatively easy to adopt and implement, in order to be routinely utilized by ED staff.

No international standard for recording presenting complaints exists, and in many EDs, recording is not systematic. Even though the need for a systematic way to record presenting complaints was raised over a decade ago by Aronsky and colleagues [7], studies around the world report vast use of free-text fields and a general lack of a structured way to record presenting complaints (Australia [8,9]; US [10]; Finland [11]). The Canadian ED Information System (CEDIS) Working Group’s Presenting Complaint List [5,12] linked to the Canadian Triage and Acuity Scale (CTAS) has achieved good coverage in Canadian EDs. Other large international triage systems (ATS, MTS, ESI) do not provide structured classifications for PCs. Other than CEDIS, there are few other classifications presented in literature. Aronsky et al. [7] aimed at developing a generally applicable set of coded chief complaints for EDs and their study resulted in a list of 54 presenting complaints. Other classifications are presented in relation to single studies [3,8,13-17].

In contrast to fragmentation of presenting complaint classifications in EDs, there are established classifications for ambulatory care. Reason for Visit Classification (RVC) is used by the Centres for Disease Control and Prevention (CDC) and for the annually reported National Hospital Ambulatory Care Survey (NHAMCS) in United States [18,19]. The International Classification for Primary Care (ICPC-2) is used for both diagnostic classification in family practice and primary care, and classification of ambulatory patients’ Reasons for Encounter. WHO has accepted ICPC-2 within the WHO’s Family of International Classifications (FIC), mainly as a method of encounter classification. ICPC-2 has been used in few studies within emergency services [20] and EDs [21,22]. However, neither RVC nor the ICPC-2 is ideal for use in EDs. Both systems are designed for primary care office hours and they each include several hundred complaints. (RVC includes 770 different complaints and the shortened version of the ICPC-2 has 687 codes for RFEs.)

The aim of the current study was to investigate whether the ICPC-2 could be adjusted to an ED presenting complaint classification, and thus, achieve an ED classification compatible with other areas of health care using the ICD-10 and the ICPC-2. The main reason for selecting the ICPC-2 was to employ an already internationally established classification as a foundation for the new classification. This study presents a structured classification for adult patients’ ED presenting complaints based on ICPC-2 classification. The authors developed the ED presenting complaint classification by using free text analysis of 40,610 visits in one ED and by collecting presenting complaint data, using the ICPC-2, from 2,400 visits in another ED. Both EDs are responsible for primary care and special care urgent and emergent patients. An expert panel finalized the classification and the authors of this study implemented the new system in the Jorvi ED. This paper discusses the results.

### Terminology

The presenting complaint in EDs refers to a professional interpretation of the symptoms or condition that made the patient seek emergency care. Presenting complaint is a term more established in Europe and Canada and its counterpart term in the U.S. is chief complaint (CC). Other terms sometimes used/mixed include Reason for Encounter (RFE), Reason for Visit, Presenting Problem, Problem on Admission, and Reason for Presenting. RFE refers to the pure reason for seeking medical advice, not including expert interpretation.

**Figure 1:**
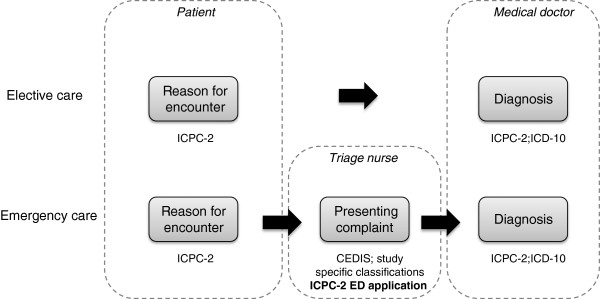
Terminology and classification systems.

Figure 1 illustrates the use of presenting complaint terminology and related classification systems. A diagnostic classification is necessary in both elective and emergency care. Therefore, the ICD-10, with a broad diagnostic range, is used in both situations. The ICPC-2 was developed for diagnostic classification in family practice and primary care and it is based on the prevalence of health problems in primary care. The term patient’s perspective represents the demand for care of that patient, and using the ICPC-2, it can be recorded as the reason for encounter both in elective and emergency care.

In emergency care, the triage nurse assesses the patient and makes decisions of urgency and requisite resources at the beginning of visits. In elective care, there is no need for the triage process or for the recording of the presenting complaint during the patient’s visit. The presenting complaint should not be confused with RFE, although it is based partly on RFE.

## Methods

### Study design

**Figure 2:**
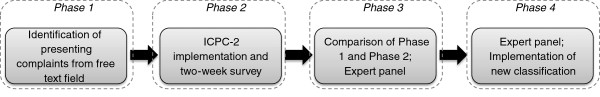
Overall study design.

The study included four phases and the overall design is illustrated in Figure 2. The objective of the first phase was to identify presenting complaints from free text fields of IT systems and to classify the presenting complaints into intuitive groups using one year data of an ED. Free text fields allowed identification of presenting complaints, as they were not biased by a classification system.

In the second phase, the objective was to implement the ICPC-2 classification in ED. As the ICPC-2 is an established classification system, mostly used in primary care diagnosis classification, the authors wanted to test its suitability for ED environments. The presenting complaints were collected with a two-week survey.

The third phase focused on creating an ICPC-2 based presenting complaint classification suitable for use in ED environments, and subsequently, in phase four to implement it in ED environments. The resulting classification system was based on the results of the first two phases and evaluated by an expert panel.

The study conducted the first phase in the Hyvinkää Hospital ED and the three others in the Jorvi Hospital ED. The following section presents both study sites and gives a more detailed description of each study phase.

### Study setting

In Finland, 24/7 emergency services are mainly centralized to joint emergency units in charge of urgent and emergency services for special and primary health care. Two emergency units from Helsinki University Hospital District (HUCH) participated in this study, the Hyvinkää Hospital ED and the Jorvi Hospital ED.

The Hyvinkää Hospital ED serves a population of 185,000 inhabitants and the ED has 49,700 visits/year (2008). The Jorvi Hospital ED serves a population of 295,000 and has 62,500 visits/year (2010). During daytime hours, the health centres in the both regions also treat urgent primary care patients. Hyvinkää Hospital situates 50 kilometres, and Jorvi Hospital 15 kilometres, from the main University Hospital clinics in Helsinki. Patients with multiple injuries, candidates for thrombolytic therapy for stroke, and patients with an ST-elevation myocardial infarction were excluded from the study because according to HUCH policy, they were forwarded to the speciality emergency departments of the HUCH hospitals in Helsinki directly, by emergency services. In addition, childbirths were excluded from the study.

### Phase 1: identification of presenting complaints from free text field

In the first phase, the objective was to identify presenting complaints of an ED using recordings from free text field. At Hyvinkää, the patients' presenting complaints are recorded in a free text field in the information system. If the patient comes without a referral, the presenting complaint is assessed by a nurse, based on the reason for the visit stated by the patient and possible information given by emergency services. If a patient has a referral, the presenting complaint is taken from the referral and entered by the department secretary. All presentations to the Hyvinkää ED during 2008 were included in the study.

Typically, if a patient is transferred from a general practitioner to a physician in specialized medical care because of a consultation, a presenting complaint is entered in the system. However, this study used the original reason for encounter entered when the patient first came to the emergency clinic.

The data were reworked by selecting the presenting complaint for each patient in the free text of the field. If several reasons for encounter were entered for a single patient, the one requiring the most urgent care was selected. The prioritised reasons were shortness of breath, chest pain, and abdominal pain, respectively. Functions in the spreadsheet application MS Excel 2007 were used to select the reasons for encounter. The data rework was done by RM and TM.

In most of the cases, obvious presenting complaints could be picked up from the free text, and therefore, grouping of the synonyms and trimming of the different writing formats comprised the majority of the work. Unclear presenting complaints were discussed case by case, and the reason selected was based on a clinician's assessment (RM). If the presenting complaint could not be determined from the field, or if the field contained just individual symptoms, the encounter was excluded from the list.

### Phase 2: ICPC-2 implementation and two-week survey

In the second phase, the objective was to implement the ICPC-2 classification to ED and evaluate its suitability to ED use. This phase was carried out in the Jorvi Hospital ED and data were collected from a survey. In Jorvi, ICPC-2 classification was already familiar, because visits to primary care had been recorded by triage nurses using ICPC-2 since 2007. However, the use of classification had not been systematic, and the concept of presenting complaint and diagnosis had been mixed. Researchers administered the survey over a continuous 14-day period, from 8:00 am Monday, November 15 to 8:00 am Monday, November 29 2010.

The presenting complaints were recorded on a paper-format questionnaire at the point of triage using ICPC-2 codes. To help staff record different ICPC-2 codes for presenting complaints, a two-page summary of codes was available at the triage facility. The two-page document is available in several languages on the WONCA website (http://www.globalfamilydoctor.com/wicc/) and it includes 687 different codes. Staff was allowed to record several presenting complaints for a single patient. However, the data analysis used only the most urgent presenting complaint.

### Phase 3: development of new ICPC-2 based ED application

In the third phase, the objective was to develop a new ICPC-2 based ED application for recording the presenting complaints. The results of the first two phases were analysed and compared to see how case mix and presenting complaints differed between the Jorvi ED and the Hyvinkää ED. Jorvi’s ICPC-2 based presenting complaints were coded to respond to the categories of the Hyvinkää ED’s study. The presenting complaints from both sites were used for the frequency comparison.

Using the data of these two EDs and experiences of ICPC-2 use in Jorvi the expert panel discussions defined the new classification for use in the ED. The panel consisted of three senior medical doctors and one nurse. TM made notes and participated to the discussion. The expert panel first reviewed the results of phases 1–3 and used the ICPC-2 based classification system. The panel also made use of other presenting complaint lists [5,12,16,17] where it was suitable and the panel members modified the ICPC-2 presenting complaint list based on the discussions. The most important criteria in selecting presenting complaints were prevalence, urgency and possible streaming to different patient pathways.

The short, two-page version of the ICPC-2 classification already included 687 RFEs; therefore, in most cases, the panel grouped overly detailed complaints into one more general group.

To preserve compatibility with the original ICPC-2, the original codes remained. However, complex codes, such as F01-F04, F13-F16, and F28-F29 would be difficult for staff to use, and most of the IT-systems could not make such an exception. Therefore, in implementation, they were changed for the Jorvi ED by using only the numerical first code of the group (in the previous example of F01, F02, F03, and F04, the code would be F01).

Presenting complaints of patients with multiple injuries, candidates for thrombolytic therapy for stroke, and patients with ST-elevation myocardial infarctions who were originally excluded from the study were taken into consideration in the new classification.

### Phase 4: implementation of ICPC-2 ED application

In the fourth phase, the objective was to implement the new ICPC-2 based ED application to Jorvi ED and to test the suitability of the classification. Before implementation of the new classification system, briefing sessions were organized for the staff. Most of the staff was familiar with the original ICPC-2 list, which made the adoption easier. Feedback from the staff was collected in weekly routine staff meetings by the head nurse in open discussions, and confidentially by a feedback box, and with one-to-one interviews conducted by RM. The staff was encouraged to give feedback from every case were they had difficulties in identifying easily an appropriate code. After two weeks use of the ICPC-2 ED application, a series of interviews captured staff experiences with the system. In total, 12 persons were interviewed one-to one by RM. Each nurse was asked the following three questions:

Question 1. Are you satisfied with the new classification?

Question 2. Have you had patients with presenting complaint which you have had difficulties to classify? If yes, please specify.

Question 3. Have you some suggestions for further development of classification or other comments?

## Results

### Results from phase 1: identification of presenting complaints from free text field

There were 49,700 patient presentations in 2008 in the Hyvinkää ED and 40,610 of them were adults (16 or over). Complete data were available in 35,334 (87,0%) patient encounters and used in the presenting complaint analysis.

The data categorization resulted in a list of 65 presenting complaints. There were 5,051 visits where the presenting complaint could not be determined. Out of these, 1,516 came for control visit due to recent visit in the ED and had only limited information about presenting complaint. In few cases the presenting complaint was due to rare disease or was related to very specific symptoms and in such a case it was not meaningful to form they own groups. The rest of the visits had either non-comprehensible or not presenting complaint related value in the free-text field. In addition, there were 225 empty presenting complaint fields. Therefore, 5,276 encounters were excluded - 13.0% of all visits. There were 9,300 patients (18.8% of all visits) with a referral.

Table 1 presents the presenting complaints used in the Hyvinkää ED and their frequencies.

**Table 1:** Presenting complaints and frequencies in Hyvinkää ED

**Presenting complaint**	**Amount**	**Percentage**	**Presenting complaint**	**Amount**	**Percentage**
Musculoskeletal symptoms/complaints	8596	21,17%	Blood pressure related problem	198	0,49%
Abdominal pain	3084	7,59%	Intravenous antibiotic infusion	183	0,45%
Upper respiratory infection/throat symptom	2862	7,05%	Bronchus/lung related symptom	182	0,45%
Shortness of breath	1567	3,86%	Allergic reaction	163	0,40%
Chest pain	1503	3,70%	Rash	149	0,37%
Diarrhoea/vomiting	1492	3,67%	Blood and blood forming organs	127	0,31%
Cardiac arrhythmia	1206	2,97%	Nose bleed	123	0,30%
Back symptom	1161	2,86%	Icterus or ascites	118	0,29%
Fever	1079	2,66%	Nausea	102	0,25%
Headache	906	2,23%	Complication of medical treatment	98	0,24%
General weakness	873	2,15%	For sick leave	93	0,23%
Infection of urinary tract	823	2,03%	Haemorrhoid	77	0,19%
Eye symptom	672	1,65%	Burn injury	72	0,18%
Symptoms of venous embolism	652	1,61%	Neurological symptoms	65	0,16%
Psychological problems	609	1,50%	Pregnancy related problem	65	0,16%
Vertigo/dizziness	589	1,45%	Constipation	64	0,16%
Cough	570	1,40%	Female genital symptoms	61	0,15%
Ear symptoms	524	1,29%	Sleep disturbance	55	0,14%
Pneumonia symptoms	452	1,11%	Symptoms of chronic bowel disease	49	0,12%
Cerebrovascular disorder symptoms	393	0,97%	High value of C-reactive protein	46	0,11%
Convulsion	389	0,96%	Catheter related	34	0,08%
Urological symptoms	380	0,94%	Dental	31	0,08%
Blood test for drunk driving suspect	344	0,85%	Cramp	30	0,07%
Intoxication	299	0,74%	Trigeminal neuralgia	23	0,06%
Cancer related symptom	298	0,73%	Tremor/shivering	22	0,05%
Drug or alcohol abuse	295	0,73%	Tick	20	0,05%
Symptoms of acute sinusitis	270	0,66%	Electric shock	17	0,04%
Lump or abscess	256	0,63%	Hypothermia	16	0,04%
Skin infection	247	0,61%	Dehydration	12	0,03%
Heart related problem, other	212	0,52%	Carbon monoxide poisoning	7	0,02%
Fainting/syncope	212	0,52%	Exposure to gas	7	0,02%
Endocrine/metabolic symptoms	210	0,52%	Other	5276	12,99%

### Results from phase 2: ICPC-2 implementation and two-week survey

During the two-week survey, there were 2332 presentations at the Jorvi ED, of which 1309 (56,1%) were in primary care and 1023 (43,9%) in secondary care. A complete data set was gathered from 1837 presentations (78,8%). Incomplete data was due to missing values in patient IDs, ages and presenting complaint information. Out of 1837 visits, 1284 were adults (16 or over) and were included to the study. Table 2 presents the divisions of presenting complaints from the Jorvi ED among general categories of the ICPC-2.

**Table 2:** Use of general categories of the ICPC-2 in Jorvi ED

**General category**	**Name**	**Amount**	**Share**
-	Process codes	6	0,47%
A	General and unspecified	172	13,40%
B	Blood, Blood Forming Organs and immune Mechanism	6	0,47%
D	Digestive	172	13,40%
F	Eye	12	0,93%
H	Ear	13	1,01%
K	Cardiovascular	149	11,60%
L	Musculoskeletal	275	21,42%
N	Neurological	87	6,78%
P	Psychological	75	5,84%
R	Respiratory	141	10,98%
S	Skin	93	7,24%
T	Endocrine/Metabolic and Nutritional	10	0,78%
U	Urological	50	3,89%
X	Pregnancy, Childbearing, Family Planning	13	1,01%
Y	Female genital	2	0,16%
Z	Male genital	4	0,31%
W	Social problems	4	0,31%

**Figure 3:**
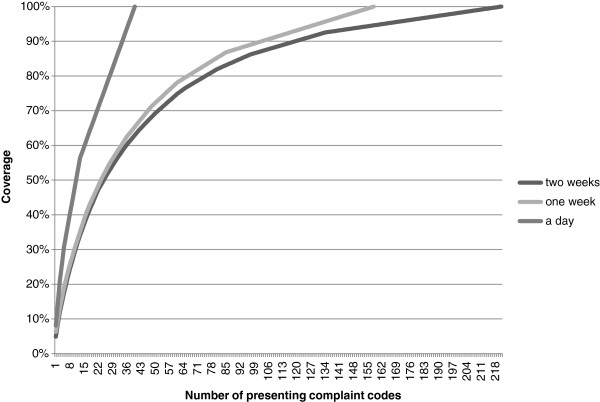
Number of presenting complaint codes from the ICPC-2 used in the Jorvi ED that achieved coverage.

At the Jorvi ED, 254 (237 for adults) out of 687 different ICPC codes were used to record presenting complaints during the two-week survey period. Figure 3 shows the coverage achieved by the number of presenting complaint codes used. The amount is dependable on volume, and therefore, different curves are given for a day (second Monday, n=72), a week (second week, n=604), and the total period (n=1284). The two-week period represented presenting complaints quite well; a longer period, with larger volume, would not have radically increased the number of presenting complaints. Coverage of 70%-90% was achieved from 50–120 different ICPC-2 based presenting complaints.

The experiences of using ICPC-2 showed in practise, that it was difficult to find a representative code for presenting complaints from ICPC-2 code list in ED. The list was far too long and included large number of codes not needed in ED. Regardless the fact that some codes require information not available at the point of triage, these codes (such as pneumonia and pulmonary embolism) were used anyway. Although the results from the phase 2 demonstrated the unsuitability of ICPC-2 classification in the raw for ED environments, it showed that ICPC-2 can be used as a basis of the ED classification. This conclusion was supported by the results of phase 3 showing that ICPC-2 classification include almost all the presenting complaints revealed in Hyvinkää data in phase 1.

### Results from phase 3: development of new ICPC-2 based ED application

Table 3 illustrates differences in the most common presenting complaints between the Jorvi ED and the Hyvinkää ED. Presenting complaints of both EDs are relatively close to each other. The biggest differences in Hyvinkää, compared to Jorvi, are in presenting complaints related to trauma mechanisms such as electric shock or carbon monoxide poisoning, for which the ICPC-2 classification does not elaborate. Some of Hyvinkää’s presenting complaints are also more diagnoses-based, such as pneumonia and urinary tract infection. In addition, in the Hyvinkää ED, the triage nurse sometimes recorded that patients came simply for a doctor’s certificate of sick leave. In Finland, most organizations allow sick leaves of 2–3 days without a certificate, but EDs are often used for sick leaves exceeding that because of the convenience of access.

**Table 3:** Top 20 presenting complaints in Hyvinkää ED and in Jorvi ED

**Top 20 presenting complaints in Hyvinkää ED (Year 2008)**	**Share**	**Top 20 presenting complaints in Jorvi ED (two weeks, Nov-2010)**	**Share**
Musculoskeletal symptoms/complaints	21,17%	Musculoskeletal symptoms/complaints	20,43%
Abdominal pain	7,59%	Abdominal pain	8,38%
Upper respiratory infection/throat symptom	7,05%	Psychological problems	4,11%
Shortness of breath	3,86%	Shortness of breath	3,89%
Chest pain	3,70%	Chest pain	3,89%
Diarrhoea/vomiting	3,67%	General weakness	3,82%
Cardiac arrhythmia	2,97%	Fever	3,75%
Back symptom	2,86%	Skin infection	3,31%
Fever	2,66%	Back symptom	3,23%
Headache	2,23%	Upper respiratory infection/throat symptom	3,09%
General weakness	2,15%	Cardiac arrhythmia	2,87%
Infection of urinary tract	2,03%	Headache	2,20%
Eye symptom	1,65%	Diarrhoea/vomiting	2,06%
Symptoms of venous embolism	1,61%	Infection of urinary tract	1,76%
Psychological problems	1,50%	Convulsion	1,25%
Vertigo/dizziness	1,45%	Vertigo/dizziness	0,96%
Cough	1,40%	Ear symptoms	0,96%
Ear symptoms	1,29%	Eye symptom	0,96%
Pneumonia symptoms	1,11%	Female genital symptoms	0,88%
Cerebrovascular disorder symptoms	0,97%	Fainting/syncope	0,73%

The classification of presenting complaints produced from the Hyvinkää ED’s free text fields classifies all musculoskeletal symptoms and complaints under one group. Such a generic group becomes too large, covering over one fifth of all the presenting complaints.

### Results from phase 4: implementation of ICPC-2 ED application

The staff experiences about implemented ICPC-2 ED application are described in the following.

Question 1. Are you satisfied with the new classification?

All interviewed nurses answered yes.

Question 2. Have you had patients with presenting complaint which you have had difficulties to classify? If yes, please specify.

All nurses answered that the suitable code was found for all presenting complaints. Two nurses reported that if a patient has many complaints, it is difficult to decide the main presenting complaint. One nurse asked, if it could be possible to get a code for pneumonia as a presenting complaint. Information of using the most urgent presenting complaint was further addressed to triage nurses as well as the problem of too early decision of possible diagnosis.

Question 3. Have you some suggestions for further development of classification or other comments?

Four nurses asked for more feedback of the results of analysis of presenting complaints. They found it important to get reports to motivate the recording.

In the weekly staff meetings people felt that the new classification was easy to use and staff members found all the presenting complaints with no difficulties except the cases with many complaints. The staff was satisfied with the new system. The original ICPC-2 list had been in use in the Jorvi ED, and therefore, the staff compared the new list to the original ICPC-2 list. They had complained that the original ICPC-2 list was too long and that the right presenting complaints were difficult to locate. As the new list was based on the ICPC-2 list, the staff felt immediately familiar with the codes. They also felt that the length and groupings were improvements to the previous list.

The feedback from the staff generated still a few adjustments regarding hyperglycaemia and delirium, which are included in final list in Table 4. The final list includes 89 presenting complaints. The list is now in routine use in Jorvi ED and the recording percent is high (over 95 %).

**Table 4:** ICPC-2 ED application

**ICPC-2 codes included**	**Name**	**ICPC-2 codes included**	**Name**
**General**		**Neurological**	
A01	Pain general/multiple sites	N01, N03	Headache; Pain face
A03	Fever	N05-06	Sensation disturbance
A04-A05	Weakness/tiredness general, feeling ill	N07	Convulsion/seizure
A06	Fainting/syncope	N17	Vertigo/dizziness
A07	Coma	N18	Paralysis/weakness
A80-A81	Trauma/injury NOS, multiple trauma	N19	Speech disorder
A87	Complication of surgical or other treatment	N29	Delirium
A88	Adverse effect physical factor	**Psychological/Toxic effects**	
A91	Abnormal result investigation NOS; Hyperglycaemia	P01	Feeling anxious/nervous/tense
A92	Allergy/allergic reaction NOS	P02	Acute stress reaction
A96	Death	P03	Feeling depressed
**Digestive**		P15-P16	Alcohol abuse
D01-D02, D06	Abdominal pain/cramps general	P18	Medication abuse
D03	Heartburn	P19	Drug abuse
D04-D05	Rectal/anal pain or itching	P20	Memory disturbance
D09-D10	Nausea, vomiting	P29	Psychological symptom/complaint other
D11, D18	Diarrhoea	P77	Suicide/suicide attempt/suicidality
D12	Constipation	A84	Poisoning by medical agent
D13	Jaundice	A86	Toxic effect non-medicinal substance
D14	Haematemesis/vomiting blood	**Respiratory**	
D15-D16	Melaena	R01-R04	Shortness of breath, dyspnoea, pain respiratory system, wheezing, breathing problem
D19	Teeth/gum symptom/complaint	R05	Cough
D20	Mouth/tongue/lip symptom/complaint	R06	Nose bleed
D25, D29	Abdominal distension	R07-R21, R28-R29, R74	Nose/sinus/throat/voice symptom/complaint
Eye		R24	Haemoptysis
F01-F03, F13-F16, F29	Eye symptoms/complaints	**Skin**	
F04-05	Visual disturbance	S01, S02,S04-S08, S99	Pain/tenderness of skin; Pruritus; Lump/swelling; Rash
**Ear**		S10-S11	Boil/carbuncle, skin-infection posttraumatic
H01-H05, H13, H29	Ear symptoms/complaints	S12	Insect bite/sting
**Cardiovascular**		S13	Animal/human bite
A11, K01-K03	Chest pain, pressure/tightness of heart	S14	Burn/scald
K04-K05	Palpitations/ awareness of heart; Bradykardia; Irregular heartbeat	S15	Foreign body in skin
K07	Swollen ankle/oedema	S18	Laceration/cut
K29	Cardiovascular symptom/complaint other, high blood pressure	**Endocrine/Metabolic**	
**Musculoskeletal**		T11	Dehydration
L01	Neck symptom/complaint	T27	Hypoglycaemia
L02-L03	Back symptom/complaint	**Urological**	
L04	Chest symptom/complaint	U01, U02, U07, U14	Dysuria/painful urination
L05	Flank/axilla symptom/complaint	U06	Haematuria
L07	Jaw symptom/complaint	U08	Urinary retention
L08	Shoulder symptom/complaint	**Pregnancy**	
L09	Arm symptom/complaint	W03	Antepartum bleeding
L10	Elbow symptom/complaint	**Female genital**	
L11	Wrist symptom/complaint	X01, X03, X08, X09, X12, X14-X17	Female genital symptoms
L12	Hand/finger symptom/complaint	X18	Breast symptoms
L13	Hip symptom/complaint	**Male genital**	
L14	Leg/thigh symptom/complaint	Y01-Y06	Male genital symptoms
L15	Knee symptom/complaint	**Social problems**	
L16	Ankle symptom/complaint	Z25	Assault/harmful event/ problem
L17	Foot/toe symptom/complaint	Z29	Social problem
L18-L19	Muscle pain; Muscle symptom/complaint NOS	**Process codes**	
		−50	Medication/prescription/renewal/injection
		−54	Repair/fixation-suture/cast/prosthetic device

## Discussion

This study created a structured complaint classification suitable for ED use and implemented it to practice. The classification is based on two stand-alone studies; one focused on identifying presenting complaints from data of 40,610 ED presentations and the other on a two-week survey while using the ICPC-2 as a classification for recording presenting complaints. The study produced a classification based on the ICPC-2, including 89 presenting complaints. The results from implementation were positive and encouraging and the classification is currently in routine use in the Jorvi ED.

Two major factors motivated the selection of the ICPC-2 as the foundation of the new classification. Firstly, the ICPC-2 is symptom-oriented. In the triage process, patients have no predefined diagnoses per se, and the triage nurse’s should base his/her interpretation of urgency and requisite resources on symptoms. Secondly, the ICPC-2 is compatible with the ICD-10. The ICPC-2 is a part of WHO’s Family of International Classifications (FIC) and all of the complaints can be traced back to related ICD-10 codes. However, the ICPC-2 has too much detail for effective use in EDs.

A short list of presenting complaints is simple and reliable, but if the list is too short, it does not present enough information. When the list is long, the specificity is higher but the system is complex and data analysis becomes difficult. With a long list, shortened modifications emerge and their use jeopardizes data comparison. ED-specific presenting complaint lists range between 33 [16] and 165 [12] in the number of different codes. The current study indicates that a list of 89 presenting complaints is well suited for ED use. In triage, time for recording codes is scarce; the system has to be easy to use. Obtaining presenting complaint information from a free text field is very time-consuming and not suitable for routine use.

Unlike the ICPC-2 based classification of this study, Canadian CEDIS classification is ED-specific. Although many presenting complaints are similar in both lists, there are remarkable differences. The main difference in this study’s shorter list is that the presenting complaints of many organs, such as ear, eye, or gynaecological symptoms, are not divided into as many subclasses as those in the CEDIS; patients in these subclasses have the same pathways and use the same resources in EDs. However, the study’s list included more subclasses to code traumas and symptoms of the extremities; symptoms of the ankle, knee, and hip have their own codes. Rare but urgent cases, such as periorbital oedema, were omitted because the inclusion of one such item would soon increase the list with other equally urgent, but rare, symptoms. One must keep in mind that almost all presenting complaints can include emergent cases.

In some cases the CEDIS list had more interpretation of the symptoms than did the list in the current study. The study list, for example, listed hyperventilation with dyspnoe to prevent premature conclusions of possible diagnoses. Hyperventilation can be a harmless symptom, but it can also be a symptom of ketoasidosis or pulmonary embolism. This same principle led to the decision to keep chest pain as one presenting complaint without trying to divide it into more or less specific cardiac features. This is important because a cardiac event is difficult to diagnose [23].

The ICPC-2 was quite easily modified and suites the classification of ED presenting complaints well. Many codes for the ED list were combined from two or three codes of the ICPC-2. However, some symptoms had to be modified from the ICPC-2. Hyperglycaemia is under abnormal investigation results in the ICPC-2, while hypoglycaemia has its own code and allergic reaction, which the new list had to include despite its features of diagnosis and the fact that it is coded as a diagnosis in the ICPC-2.

Classification of presenting complaints does not remove the need for using free text communication in EDs, it is highly important and codes or rules should not restrict its use. In the Jorvi Hospital ED, the presenting complaint code is not used for communication at all; it is registered only for purposes of data analysis and quality studies and free text is used for communication.

Decisions made in triage regarding urgency and tracks have significant effects on the duration and the quality of the care process. The presenting complaint is one of the most important variables affecting these decisions. The variety of patients entering EDs is wide, and to evaluate triage performance and care quality, divisions between different patient groups are necessary. Often studies concerning EDs make divisions and give treatment recommendations based on diagnoses or treatments but such information is not available at the point of triage. Presenting complaints provide more relevant divisions for studying quality, process, and outcomes of care; for such research, structured information regarding presenting complaints is essential.

In addition, routine research in the classification of presenting complaints in ERs enables several practical data usage possibilities. Presenting complaint information, for example, aids demand and capacity planning, streaming, and patient flow control, quality control, and benchmarking of performance.

### Limitations

Although the studied EDs represent medium-sized joint EDs, which are typical to Finnish healthcare system, there may be need for customization and for more detailed presenting complaints classification in highly specialized units. Moreover, the new classification was tested only in one ED and to ensure reliability and validity with different case-mixes further studies may be needed.

It should be noted that the evaluation method of new classification is not strictly following any qualitative method and is not reported according to any qualitative standard.

All classifications need continuous improvement. Therefore, our study classification is not suitable for broad use without centralized national or international actor for development. The study focused only in adult patients and the classification is not directly generalizable to presenting complaints of children.

## Conclusions

The ICPC-2 classification can be easily modified for use in EDs by decreasing the number of codes. The use of structured classification for recording presenting complaints in EDs helps to compare and improve EDs, both nationally and internationally. Recording presenting complaints provides information from ED case-mixes and helps in the planning and control of patient flows.

It is essential that future research develops a similar, ICPC-2 based presenting complaint list for pediatric emergency care. Further validation studies should also be conducted to the classification in different environments using standard evaluation protocols such as COREQ [24].

The authors recommend the use of the ICPC-2 based list for recording ED presenting complaints; the new list with 89 presenting complaints was more convenient and easy to use. The classification is easily transferable to different EDs, although such a classification needs to be developed and improved continually. To ensure data comparability, all such improvement modifications should be centralized, either by national actor or by an international organization willing to take the classification forward, such as the WONCA International Classification Committee.

## Competing interests

The authors declare that they have no competing interests. TM and PT are employed by Nordic Healthcare Group, NHG. NHG is a commercial company that focuses on healthcare and welfare industries and designs models to enhance productivity, cost-effectiveness and process quality. The business is based on research and has employees in Stockholm, Sweden and Helsinki, Finland.

## Authors’ contributions

TM designed the study, conducted the data analysis for Hyvinkää and Jorvi data, and drafted the manuscript. OH helped in the data collection (Jorvi and expert panel) and revised the manuscript. PT helped in the data analysis and revised the manuscript. RM co-designed the study, organized the data collection (Hyvinkää, Jorvi, and expert panel), performed the interviews, and helped to draft the manuscript. All authors read and approved the final manuscript.
